# Inclusive Finance, Environmental Regulation, and Public Health in China: Lessons for the COVID-19 Pandemic

**DOI:** 10.3389/fpubh.2021.662166

**Published:** 2021-04-12

**Authors:** Xia Liu, Suqin Guo

**Affiliations:** ^1^School of International Finance and Trade, Sichuan International Studies University, Chongqing, China; ^2^Chongqing Dongnan Hospital, Southeast Hospital, Chongqing, China

**Keywords:** public health, COVID-19, inclusive finance, environmental regulation, PPML estimator

## Abstract

The slow-down of the Chinese economy and the depression in the global economy during the COVID-19 show that governments should provide stimulus packages. These policies should be inclusive in terms of financial gains. Using the panel data of 30 regions in China from 2006 to 2016, this paper uses the Poisson Pseudo-Maximum Likelihood (PPML) estimator to analyze the impact of inclusive finance on public health. The results show that inclusive finance has a significant positive effect on public health. The performance of the eastern region is significantly better than that of the central and western regions. When we consider the combined effect of environmental regulation, the improvement effect of inclusive finance on public health is still significant, and the coefficient increases in the eastern region. Similarly, there is also a significant improvement effect in the central and western regions. Our findings reveal that environmental regulation promotes the beneficial effect of inclusive finance. Therefore, it is important to improve the inclusive financial development mechanism and enhance environmental regulation intensity for solving public health issues. Lessons related to the COVID-19 pandemic are also discussed.

## Introduction

The wheel of history has left two deepest marks on China in the great journey of more than 40 years of reform and opening up: the sustained and rapid economic take-off and the sharp deterioration of human health and the ecological environment. Pollution makes human beings face huge health costs and survival threats. It causes rapid depreciation of human health capital, which constitutes an important source of economic and social development inequality in the world (1). Health risks caused by environmental pollution require environmental regulations, which will have positive effects on public health. This paper examines the effects of the inclusive finance on public health in China. During the COVID-19, it is observed that the low degree of inclusiveness cannot effectively meet the real economy's needs and market entities for financial development and financial innovation. The slow-down of the Chinese economy and the depression in the global economy during the COVID-19 show that governments should provide stimulus packages. These policies should be inclusive in terms of financial gains.

To deal with this thorny problem, environmental regulation, as the preferred tool and weapon of all countries, came into being (2). In 2007, China formulated the first programmatic document in the environment and health, *the National Environment and Health Action Plan (2007-2015)*. In 2014, the eighth meeting of the Standing Committee of the 12th National People's Congress voted and passed the amendment to the environmental protection law, completing the first amendment to the environmental protection law. However, the challenge brought by pollution far exceeded the imagination and expectation of all sectors of society. According to the *Environmental Green Paper: China's Environmental Development Report (2016-2017)*, the Beijing Municipal Government twice issued the early red warning of heavy air pollution in just over a month from 2015 to the beginning of 2016. This is the first time Beijing launched the early red warning after introducing the four-level response policy of air pollution in 2013. Do these facts mean that China's environmental regulation is invalid? This is why we are exploring the internal relationship and mechanism between environmental pollution, environmental regulation, and residents' health is of great help to recognize and measure the high cost of environmental pollution and highlight the necessity and importance of formulation and implementation of government environmental regulation policy. Therefore, it is urgent to speed up the formulation and promulgation of relevant environmental regulation policies and efficient environmental pollution control. More importantly, it is necessary to strengthen the overall awareness among regions, enhance the exchange and cooperation between governments in the formulation and implementation of pollution control and environmental regulation to achieve the good effect of “1 + 1 > 2.”

Since China's reform and opening up to finance and trade, financial repression has resulted in the abnormal wealth distribution mechanism of the residents' sticking back to the enterprises, meaning that the financial resources have not been effectively allocated. As inclusive finance can effectively optimize the allocation of financial resources and adjust the return on capital to a reasonable range, inclusive finance has become an important means to improve countries' financial systems in the world. Inclusive finance refers to improving financial infrastructure, reducing transaction costs, and effectively expanding the scope and population of financial services. In this sense, this paper uses panel data of 30 provinces from 2006 to 2016 as research samples. It adopts Poisson's pseudo maximum likelihood estimation method (PPML) to analyze the impact of inclusive finance on Global Trade Finance Program GTFP based on environmental regulation. It is of great practical significance to reveal the combined effect of inclusive finance and environmental regulation on public health, which is conducive to improving the quality and efficiency of medium and long-term social public welfare. This research is the first study to analyze the effects of financial inclusion on public health in China using the region-level dataset to the best of our knowledge.

The rest of the paper is organized as follows. Section Literature Review reviews the related literature. Section Empirical Model and Variable Selection explains the empirical model and variable selection criteria. Section Empirical Results and Analysis provides the empirical findings. Section Conclusions concludes.

## Literature Review

In terms of the definition of inclusive finance and the construction of indicators, Beck et al. (3) first defined inclusive finance. The authors constructed the indicators from two dimensions: availability and usability. Sarma (4) further improved and developed the content of inclusive finance. He constructed a comprehensive index of inclusive finance from three dimensions of financial penetration, availability and usability. In terms of the advantages of inclusive finance, Bruhn and Love (5) suggested that compared with traditional finance, inclusive finance has a wider coverage, which can also optimize the allocation of capital and create preconditions for the upgrading of industrial structure and the development of science and technology. At present, scholars have provided little research on the application of inclusive finance. These papers mainly focused on the impact of inclusive finance on labor income and industrial structure. Huang and Zhang (6) analyzed the regional data from China and found that inclusive finance had a significant positive impact on labor income. With the improvement of inclusive finance, China's share of labor income would also rise.

Theoretically speaking, environmental regulation will increase the expenditure on pollution control and investment of new technology. Although internal financing can alleviate some financing constraints, it is necessary to use the financial system's external financing due to large capital demand, tight governance time, and insignificant short-term return. A higher level of financial development is conducive to environmental regulation and the realization of a “win-win” between environmental governance and technological innovation (7) since financial development can restrain the economic fluctuation caused by environmental regulation (8). At the same time, environmental regulation can significantly improve the quantity and quality of environmental information disclosure, provide information screening for financial institutions, promote green credit, boost green technology innovation, and have a significant positive impact on public health (9). Besides, environmental regulation also forces financial reform and guides financial institutions to develop the blue ocean market of environmental protection. It can be seen that financial development, environmental regulation and their synergistic interaction play an important role in public health.

There are two main research threads on environmental regulation and public health: one is to assess the health risks caused by environmental pollution and demonstrate the adverse health effects of pollution. According to the research purpose, Cropper (10) introduced environmental pollution into the model as an important variable that significantly affected human health and made gradual and continuous improvement based on Grossman's health production function. Albertini et al. (11) explored the reasons behind the phenomenon that residents in heavily polluted areas were generally faced with accelerated depreciation of health stock and found that despite age, environmental pollution is a major cause of the further increase of health depreciation rate accelerated decrease of health stock. Ebenstein (12) explained the incidence rate of digestive tract cancer in China based on the new perspective of river water pollution. The study found that every 1% decrease in water quality will cause nearly 10% of digestive tract cancer incidence. The pollution of river water has become a hidden tumor that endangers our residents' health and threatens their survival. Saygin et al. (13), based on the Grossman model, empirically analyzed the substantial harm of PM10 and SO_2_ to residents' health from a micro perspective. The research showed that it was particularly urgent to use all effective measures to effectively control air pollution and curb the continuous decline of air quality. Secondly, to deal with the worsening situation of environmental conditions and residents' health, this paper discussed how to formulate environmental policies scientifically and reasonably and further analyzed the environmental performance and economic impact of this government behavior. Based on the data of Dublin, Ireland, Boogaard et al. (14) verified that the formulation and implementation of environmental regulation policies were indeed conducive to the improvement of environmental quality, greatly reducing the emissions of pollutants, thus highlighting the importance and necessity of government environmental regulation. Cao et al. (15) combined the Arrow-Romer production function with the Grossman utility function and used China's cross-regional data to explore environmental regulation policy's economic impact. The study found that: because health investment might crowd out physical capital, excessive health investment might negatively affect economic growth.

By summarizing the existing literature, we can find that scholars have done comprehensive research on the health effect of environmental pollution and the economic effect of environmental regulation. Still, there is almost no literature on the internal relationship and mechanism among inclusive finance, environmental regulation and public health. Firstly, few scholars theoretically analyzed the mechanism of financial development, environmental regulation and their synergy on public health and verified the collective impact based on empirical methods. Secondly, financial development and environmental regulation are often measured by a single indicator, which cannot effectively measure and reflect them (16, 17).

## Empirical Model and Variable Selection

### Model Setting

The error term violating the assumption of the same variance may raise the problem of endogeneity bias. However, the dynamic panel data estimators measure the short-term impact of related variables on the total factor productivity rather than the long-term relationship. Thus, this paper uses the Poisson Pseudo-maximum Likelihood (PPML) estimators proposed by Silva and Teneyro (18, 19) as regression estimation. Silva and Teneyro (18, 19) proposed the PPML estimation method to solve the above two problems and exemplified the gravity model used in international trade research. Note that the PPML estimator is scale-invariant since we have used the log-transformed dependent variable (20). Besides, the PPML method can solve a possible endogeneity bias. Simultaneously, Arvis and Shepherd (21) confirmed the effectiveness of the method. Based on the PPML estimation method, our paper examines the impact of inclusive finance and environmental regulation on public health in 30 provinces except Tibet in 2006–2016 in China. The benchmark model is constructed as following the control variables in previous papers [see, e.g., (22)]:

(1) $$\begin{array}{l} Hd_{i,t}=\alpha_{0}+\alpha_{1}\ln\;ifi_{i,t}+\alpha_{2}\ln\;envi_{i,t}+\alpha_{3}\ln\;pgdp_{i,t}\\ \;+\alpha_{4}\ln\;urban_{i,t}+\alpha_{5}\ln\;medi_{i,t}+\alpha_{6}\ln\;fiscal_{i,t}+\varepsilon_{i,t} \end{array}$$

Where *i* and *t* represent provinces and years, respectively. *Hd* stands for public health; *ifi* represents the inclusive finance. In terms of control variables, *envi*stands for environmental regulation [see, e.g., (23)]; *pgdp* is GDP per capita [see, e.g., (24)]; *urban* stands for urbanization rate (25); *medi* stands for medical service [see, e.g., (26)]; *fiscal* stands for proportion of medical and health expenditure [see, e.g., (24)]; ε is the random error term.

Following the spirit of Van Zoest and Hopman (27), Model (1) focuses on the impact of inclusive finance on public health. To further consider the regulatory role of environmental regulation on the impact of inclusive finance on public health, this paper constructs the following regression model based on the benchmark model:

(2) $$\begin{array}{l} Hd_{i,t}=\alpha_{0}+\alpha_{1}\ln\;ifi_{i,t}+\alpha_{2}\ln\;ifi_{i,t}*\ln\;envi_{i,t}+\alpha_{3}\ln\;envi_{i,t}\\ \;+\alpha_{4}\ln\;pgdp_{i,t}+\alpha_{5}\ln\;urban_{i,t}+\alpha_{6}\ln\;medi_{i,t}\\ \;+\alpha_{7}\ln\;fiscal_{i,t}+\varepsilon_{i,t} \end{array}$$

Where ln *ifi*_*i,t*_ * ln *envi*_*i, t*_ represents the combined effect of inclusive finance and environmental regulation. When the coefficient is significantly >0, the improvement of environmental regulation will assist inclusive finance in promoting public health; on the contrary, public health will be hindered.

### Variable Selection and Data Source

This paper will use the data of 30 provinces except Tibet from 2006 to 2016 in China as the research sample. The main data sources include provincial statistical yearbooks, China regional financial operation reports and China health and family planning statistical yearbooks. Due to the significant differences in economic development and financial development between regions in China, this paper will divide the 30 provinces into three regions: the eastern region, the central region and the western region based on the comprehensive study. The eastern region includes 11 provinces, including Beijing, Tianjin, Hebei, Liaoning, Shanghai, Jiangsu, Zhejiang, Fujian, Shandong, Guangdong and Hainan; the central region includes eight provinces, including Heilongjiang, Jilin, Shanxi, Anhui, Jiangxi, Henan, Hubei and Hunan; the western region includes 11 provinces, including inner Mongolia, Guangxi, Chongqing, Sichuan, Guizhou, Yunnan, Shanxi, Gansu, Qinghai, Ningxia and Xinjiang 11 Provinces and municipalities.

#### Inclusive Finance

This paper uses the inclusive financial index (IFI) proposed by Sarma (4) to represent. Inclusive finance includes three dimensions: financial penetration, availability and usability. The larger the index value is, the higher the level of inclusive financial development becomes. This paper cannot get financial penetration data due to data availability limitation, so we analyze it from financial availability and usability. In this paper, the number of banking outlets per 100,000 people and the number of banking services per 100,000 people are used to measure financial usability, and the weight of both is 1/2. At the same time, this paper uses the ratio of total deposits and total loans to GDP to measure financial availability, and the weight of both is 1/2. The specific measurement formula of inclusive financial index (IFI) is as follows:

(3) $$\begin{array}{l} \begin{array}{c} IFI=1-\frac{\sqrt{{\left(w_{1}-d_{1}\right)}^{2}+{\left(w_{2}-d_{2}\right)}^{2}+...+{\left(w_{n}-d_{n}\right)}^{2}}}{\sqrt{{w_{1}}^{2}+{w_{2}}^{2}+...+{w_{n}}^{2}}}\\ d_{i}=w_{i}\frac{A_{i}-m_{i}}{M_{i}-m_{i}},0\leq w_{i}\leq 1,i=1,2,...,n \end{array} \end{array}$$

The above formula *A*_*i*_ represents the real value of dimension *i*; *m*_*i*_ and *M*_*i*_ represent the minimum value and maximum value of dimension *i*, respectively; *w*_*i*_ represents the weight of dimension *i*.

#### Environmental Regulation

At present, there is no unified environmental regulation index in the academic circle, and the commonly used alternative indicators are the emission intensity of a certain pollutant, the operation cost of pollution control facilities, the income of sewage charges, etc. Besides, some scholars constructed a relevant composite index to characterize the intensity of environmental regulation. The formulation of environmental regulation is measured by the number of proposals (pieces) of the National People's Congress on environmental protection; regulation implementation is measured by the proportion of environmental pollution control investment in GDP (%) and the proportion of industrial pollution control investment in industrial added value (%); regulatory supervision is measured by the proportion (%) of sewage fee income in industrial added value.

In terms of other control variables, GDP per capita (*pgdp*) is the ratio of GDP to the number of permanent residents; urbanization rate (*urban*) is the proportion of urban population in the total population; medical service (*medi*) is health professionals per 1,000 population; the proportion of medical and health expenditure (*fiscal*) is the proportion of medical and health expenditure in financial expenditure.

## Empirical Results and Analysis

### Benchmark Regression Results

Table 1 shows the regression results. In column (1), this paper uses the static panel estimation method and then compares it with the PPML estimation results. After the Hausman test, it is found that the *p*-value rejects the original hypothesis, and then this paper chooses the fixed-effects estimation method. In columns (2)–(8), the PPML method is used to estimate and control variables are added gradually to test the robustness of the estimation results.

**Table 1:** Regression estimation results of inclusive finance on public health.

	**FE**	**PPML**	**PPML**	**PPML**	**PPML**	**PPML**	**PPML**	**PPML**
	**(1)**	**(2)**	**(3)**	**(4)**	**(5)**	**(6)**	**(7)**	**(8)**
ln *ifi*	0.149 (1.05)	0.084^**^ (2.45)	0.123^**^ (2.37)	0.128^**^ (2.42)	0.135^**^ (2.21)	0.140^*^ (1.89)	0.152^*^ (1.85)	0.156^*^ (1.75)
ln *envi*	0.256^**^ (2.34)		0.272^***^ (2.58)	0.070^***^ (1.56)	0.273^**^ (2.45)	0.275^**^ (2.48)	0.275^**^ (2.47)	0.272^**^ (2.31)
ln *pgdp*	0.053^***^ (3.02)			0.049^***^ (3.56)	0.056^***^ (3.58)	0.050^***^ (3.42)	0.059^***^ (3.21)	0.063^***^ (3.22)
ln *urban*	0.072 (1.34)				−0.126^**^ (−2.34)	−0.083^**^ (−2.08)	−0.081^*^ (−1.83)	−0.085^**^ (−1.97)
ln *medi*	0.106^***^ (2.61)					0.109^**^ (2.27)	0.108^**^ (2.40)	0.108^**^ (2.42)
ln *fiscal*	0.134^**^ (−2.21)						0.113^*^ (1.69)	0.115^*^ (1.77)
Constant	0.529^***^ (5.59)	−0.634^***^ (−5.66)	−0.633^***^ (−5.21)	−0.658^***^ (−8.51)	−0.690^***^ (−5.61)	−0.753^***^ (−6.56)	−0.724^***^ (−7.93)	−0.723^***^ (−7.55)
*R*^2^	0.856	0.513	0.505	0.513	0.520	0.526	0.525	0.531
Time-effects	Yes	Yes	Yes	Yes	Yes	Yes	Yes	Yes
Reg.-effects	Yes	Yes	Yes	Yes	Yes	Yes	Yes	Yes
Observations	330	330	330	330	330	330	330	330

***, **, * *represent the 1, 5, and 10% levels of significance, respectively. The values reported in brackets are t-statistics*.

It can be seen from the estimation results in column (1) that inclusive finance does not significantly promote public health. Although the coefficient is 0.149, it has not passed the significance test. From the results of column (2), inclusive finance shows a significant improvement in public health after regression estimation with the PPML. With the increase of control variables, the significant positive effect of inclusive finance on GTFP is relatively stable. Although the significance level decreases, it still passes the significance test at the level of 10%. Through the comparison of the estimation results of columns (1) and (2)–(8), it is noticeable if we use the regression results of the fixed-effect estimations method based on the static panel, we will come to the wrong conclusion that inclusive finance cannot promote public health.

From the results of control variables, the coefficient of environmental regulation is positive. This evidence is in line with Wagner (23). It has passed the significance level test, which shows that environmental regulation has an obvious improvement effect on public health. The coefficient of per capita GDP is positive in columns (4)–(8). It is significant at the level of 1%, which indicates that public health will be greatly improved with further economic development improvement. This result is in line with the findings of Edeme et al. (24), which show that more local pillar industries have changed from the second industry to the third industry with less pollution. The urbanization rate coefficient is significantly negative, which shows that urbanization development in China is at the environment's expense. This finding is different from previous papers. The coefficients of medical service and the proportion of medical and health expenditure are positive. Both coefficients pass the 1% significance level, which shows that the two's improvement has a significant positive role in promoting public health. This evidence is in line with Shah (26).

### Regional Regression Results

Based on the regression analysis above, this paper will divide the 30 provinces into the eastern region, the central region and the western region, and then analyze and discuss the impact of inclusive finance on public health. Table 2 is the detailed regression results.

**Table 2:** Regional regression results.

**Explanatory variable**	**Eastern regions**		**Central regions**		**Western regions**	
	**(1)**	**(2)**	**(3)**	**(4)**	**(5)**	**(6)**
ln *ifi*	0.206^**^ (2.45)	0.192^**^ (2.27)	0.105^**^ (2.03)	0.097^*^ (1.92)	0.146^*^ (1.89)	0.133^*^ (1.70)
ln *envi*		0.245^***^ (2.65)		0.232^**^ (2.42)		0.236^**^ (2.37)
ln *pgdp*		0.124^***^ (4.86)		0.160^***^ (4.21)		0.035^**^ (2.01)
ln *urban*		−0.097^**^ (−2.31)		−0.062^**^ (−2.22)		−0.058^**^ (−1.98)
ln *medi*		0.119^***^ (2.71)		0.107^**^ (2.32)		0.108^**^ (1.97)
ln *fiscal*		0.192^**^ (2.30)		0.133^*^ (1.82)		0.130^*^ (1.89)
Constant	−0.652^***^ (−5.99)	−0.641^***^ (−5.70)	−0.735^***^ (−6.31)	−0.756^***^ (−6.45)	−0.620^***^ (−5.79)	−0.608^***^ (−5.66)
R^2^	0.521	0.535	0.515	0.520	0.502	0.513
Time effects	Yes	Yes	Yes	Yes	Yes	Yes
Region effects	Yes	Yes	Yes	Yes	Yes	Yes
Observations	121	121	88	88	121	121

***, **, * *represent the 1, 5, and 10% levels of significance, respectively. The values reported in brackets are t-statistics*.

Table 2 shows significant differences in the impact of inclusive finance on public health among regions. Compared with the central and western regions, inclusive finance plays the most important role in improving public health in the eastern region. The coefficient reaches 0.192 and passes the significance level of 5%. This result shows that inclusive finance keeps a positive role in promoting public health and is higher than the national average level in Table 1 and the central and western regions in Table 2, which fully reflects the leading advantages of inclusive finance in the eastern region. Taking the number of banking outlets as an example, the average number in the eastern region is 9,043, while the average number in the central and western regions is 8,336 and 7,578, respectively. From this data, we can see that the eastern region is more prominent in financial advantages. In the central region, the coefficient of inclusive finance is 0.097; in the western region, the coefficient of inclusive finance is 0.133, and both pass the significance level test. From the comparison between the central region and the western region, inclusive finance has a greater influence on public health in the western region and a relatively smaller influence on public health in the central region, which is closely related to the long-term adherence to the “western development” policy and the stronger marginal role of inclusive finance in the western region.

### Regression Results Under the Effect of Environmental Regulation

The impact of inclusive finance on public health depends on its development and is affected by environmental regulation. We also test whether the environmental regulation can promote the significant positive effect of inclusive finance on public health. Therefore, we use model (2), which adds the multiplier of inclusive finance and environmental regulation. Because of the strong correlation between the cross multiplication term and the independent variables, the model is with the multicollinearity problem. Since the decentralization of the cross-multiplication terms can effectively alleviate the multicollinearity problem (28), this paper adopts the method of Wooldridge (28) to decentralize all the cross multiplication terms in the model (2). The regression results are shown in Table 3.

**Table 3:** Regression results under the effect of environmental regulation.

**Explanatory Variable**	**Whole Country**		**Eastern Regions**		**Central Regions**		**Western Regions**	
	**(1)**	**(2)**	**(3)**	**(4)**	**(5)**	**(6)**	**(7)**	**(8)**
ln *ifi*	0.126^**^ (2.45)	0.148^*^ (1.72)	0.213^**^ (2.45)	0.181^**^ (2.07)	0.105^*^ (1.89)	0.083^*^ (1.92)	0.125^*^ (1.72)	0.139^*^ (1.70)
ln *envi*	0.011 (1.30)	0.012 (1.29)	0.048 (1.52)	0.054 (1.60)	0.027 (1.26)	0.032 (1.42)	0.023 (1.44)	0.042 (1.37)
ln *ifi* × ln *envi*	0.281^***^ (2.74)	0.323^***^ (2.69)	0.356^**^ (2.21)	0.315^**^ (2.30)	0.292^**^ (2.06)	0.278^*^ (1.93)	0.211^**^ (1.98)	0.216^**^ (1.97)
ln *pgdp*		0.072^***^ (3.45)		0.162^***^ (4.34)		0.145^***^ (4.08)		0.036^**^ (2.30)
ln *urban*		−0.090^*^ (−1.86)		−0.109^**^ (−2.34)		−0.065^**^ (−2.34)		−0.062^**^ (−2.07)
ln *medi*		0.108^**^ (2.35)		0.121^**^ (2.14)		0.110^**^ (2.23)		0.108^*^ (1.79)
ln *fiscal*		0.123^**^ (2.39)		0.130^**^ (2.31)		0.132^**^ (1.99)		0.148^*^ (1.89)
Constant	−0.509^***^ (−7.72)	−0.662^***^ (−8.74)	−0.652^***^ (−5.99)	−0.734^***^ (−6.35)	−0.703^***^ (−5.36)	−0.714^***^ (−6.17)	−0.620^***^ (−5.79)	−0.573^***^ (−5.32)
R^2^	0.498	0.517	0.502	0.514	0.495	0.508	0.461	0.475
Time effects	Yes	Yes	Yes	Yes	Yes	Yes	Yes	Yes
Region effects	Yes	Yes	Yes	Yes	Yes	Yes	Yes	Yes
Observations	330	330	121	121	88	88	121	121

***, **, * *represent the 1, 5, and 10% levels of significance, respectively. The values reported in brackets are t-statistics*.

From the regression results in column (2) of Table 3, it can be seen that under the combined effect of environmental regulation, inclusive finance has an improvement effect on public health of the whole country, and its coefficient is greater, indicating that the environmental regulation promotes the role of inclusive finance in public health. From the perspective of regions, the combined effect of inclusive finance and environmental regulation can significantly improve public health in the eastern region and significantly improve the vast central and western regions. For the central and western regions, the combined effect of inclusive finance and environmental regulation can significantly improve public health, which shows that environmental regulation greatly promotes inclusive finance in the two regions. This result is related to catching up with and surpassing the heavy industry economy implemented for a long time in China. Due to the regional conditions and relative disadvantages of resources in the central and western regions' economic development, local governments rely more on industry to develop regional economies. Simultaneously, most of the bank's increased loans also flow to the state-owned enterprises with heavy capital, so the intermediary financial efficiency in the central and western regions is significantly lower than that in the eastern regions.

## Conclusions

Finance is an important mechanism and path to promote the development of public health in China. At present, many scholars mainly pay attention to financial deepening indicators such as financial scale and total amount, but researches on inclusive finance aiming at solving financial repression are limited. Because of its important role in green development and industrial structure upgrading, the construction of an inclusive financial system and its effect has become one of China's main financial development contents. Based on the regional data from 2006 to 2016, this paper uses the PPML estimation method to analyze the impact of inclusive finance on public health. Further, it investigates the collective impact of environmental regulation.

The results show that, on the whole, inclusive finance has a significant effect on improving public health. Besides, from the perspective of regions, inclusive finance has a stronger effect on improving public health in the eastern region than in the central and western regions. In comparison, the western region has a stronger effect on improving public health than the central region. In general, the crossover term between environmental regulation and inclusive finance is significantly positive. Its coefficient is significantly greater, meaning that environmental regulation has played an accelerating role in promoting the effect of inclusive finance. Finally, under the effect of environmental regulation, inclusive finance has a most significant improvement effect on public health in the eastern region, and the improvement effect on the central and western regions is relatively small, which is closely related to the regional economic development endowment, industrial development policy and long-term credit policy.

There are various main implications and lessons for the COVID-19 pandemic following our results. Firstly, policymakers should actively build and improve inclusive finance's development mechanism. The low degree of inclusiveness cannot effectively meet the real economy's needs and market entities for financial development and financial innovation. Simple financial deepening cannot effectively solve the structural contradictions in China's financial resource allocation. When reforming and improving the current financial system, we should pay more attention to the real economy's inclusive financial needs. Therefore, policymakers should focus on constructing an inclusive financial system and mechanism and improving financial innovation's ability to break the reform. At this stage, people's trust in policymakers will be vital during the COVID-19 (29).

Secondly, policymakers should establish differentiated inclusive financial green development mechanisms according to local conditions. There are significant differences in economic, financial conditions and resource endowment between the eastern region, the central region and the western region. More importantly, the low degree of marketization has become one of the main obstacles to inclusive finance's green effect in the middle and western regions. Therefore, they should actively build an inclusive financial green development mechanism to expand market participation and further promote the central region's green development and the western region.

Finally, government should improve financial efficiency and strengthen inclusive finance in promoting the environment. For a long time, China's credit mechanism is relatively abnormal. A large number of financial resources flow to state-owned enterprises, resulting in the overall low efficiency of financial intermediary in China, which also affects the play of inclusive finance on the improvement effect of GTFP. Therefore, to make full use of inclusive finance on green development, policymakers should take credit policy as a breakthrough to effectively improve financial intermediaries' efficiency in China. Future studies can use the indicators in our paper by updating the data until the post-COVID-19 period.

## Data Availability Statement

Publicly available datasets were analyzed in this study. This data can be found here: https://data.stats.gov.cn/.

## Author Contributions

XL: writing the original draft and econometric analyses. SG: writing the original draft and data access. Both authors contributed to the article and approved the submitted version.

## Conflict of Interest

The authors declare that the research was conducted in the absence of any commercial or financial relationships that could be construed as a potential conflict of interest.
